# Twelve-month contraceptive continuation among women initiating short- and long-acting reversible contraceptives in North Kivu, Democratic Republic of the Congo

**DOI:** 10.1371/journal.pone.0182744

**Published:** 2017-09-08

**Authors:** Sara E. Casey, Amy Cannon, Benjamin Mushagalusa Balikubirhi, Jean-Bosco Muyisa, Ribka Amsalu, Maria Tsolka

**Affiliations:** 1 RAISE Initiative, Heilbrunn Department of Population and Family Health, Mailman School of Public Health, Columbia University, New York, New York, United States of America; 2 Save the Children USA, Washington DC, United States of America; 3 Save the Children, Goma, Democratic Republic of the Congo; Yale University School of Nursing, UNITED STATES

## Abstract

**Context:**

Despite the inclusion of sexual and reproductive health (SRH) services in the minimum standards of health care in humanitarian settings, access to SRH services, and especially to contraception, is often compromised in war. Very little is known about continuation and switching of contraceptive methods in these settings. An evaluation of a contraceptive services program in North Kivu, Democratic Republic of the Congo (DRC) was conducted to measure 12-month contraceptive continuation by type of contraceptive method (short-acting or long-acting).

**Methods:**

A stratified systematic sample of women who initiated a contraceptive method 12–18 months prior to data collection was selected retrospectively from facility registers. A total of 548 women was interviewed about their contraceptive use: 304 who began a short-acting method (pills, injectables) and 244 who began a long-acting method (intra-uterine devices, implants). Key characteristics of short-acting method versus long-acting method acceptors were compared using chi-square statistics for categorical data and t-tests for continuous data. Unadjusted and adjusted Cox proportional hazard ratios were estimated to assess factors associated with discontinuation.

**Results:**

At 12 months, 81.6% women reported using their baseline contraceptive method continuously, with more long-acting than short-acting contraceptive acceptors (86.1% versus 78.0%, p = .02) continuing contraceptive use. Use of a short-acting method (Hazard ratio (HR) 1.74 [95%CI 1.13–2.67]) and desiring a child within two years (HR 2.58 [95%CI 1.45–4.54]) were associated with discontinuation within the first 12 months of use. The vast majority (88.3%) of women reported no prior contraceptive use.

**Conclusion:**

This is the first study of contraceptive continuation in a humanitarian setting. The high percentages of women continuing contraceptive use found here demonstrates that women will choose to initiate and continue use of their desired contraceptive method, even in a difficult, unstable and low contraceptive prevalence setting like North Kivu.

## Introduction

The complex humanitarian emergencies caused by armed conflict are characterized by social disruption, population displacement and the breakdown of national health systems [1, 2]. In addition to their need for shelter, food, water and primary health care, women living in humanitarian settings face many sexual and reproductive health (SRH) concerns including high risk of mortality and morbidity due to pregnancy-related causes, unintended pregnancy due to lack of information or access to contraceptive services, complications of unsafe abortions, gender-based violence and sexually transmitted infections including HIV [3]. Humanitarian assistance standards now recognize this increased risk and SRH services are included in the minimum package of care [4].

Although access to SRH services has improved in humanitarian settings, a 2012–2014 evaluation of SRH in humanitarian settings showed limited progress for contraceptive services relative to other SRH components [5]. Only 14.9% of the humanitarian health appeals from 2009–2013 requested funding for contraceptive services, the smallest share of any of the SRH components; contraceptive services subsequently received the lowest dollar amount among SRH components (US$76.3 million out of US$1.5 billion) [6]. Further, long-acting and permanent contraceptive methods were rarely mentioned in the appeals. The 2012–2014 evaluation also found that 36 of the 63 health facilities assessed in three humanitarian settings provided pills and injectables, and only 11 met the minimum quality criteria to provide long-acting contraceptives [7]. Overall, the findings of the 2012–2014 global evaluation reflected the limited attention given to the provision of contraceptive services in humanitarian settings.

Recent evidence suggests that contraceptives, including a broad range of methods, can be effectively provided and will be used by women and men in humanitarian settings [8–10]. However, no data on contraceptive continuation in these settings exists. Effective contraceptive programs not only help women to initiate methods when they desire, but also ensure that women are satisfied with their contraceptive method or help them switch to a new one, and encourage continuity of their preferred method as long as they wish. An analysis of 60 Demographic and Health Surveys (DHS) from 25 countries in Africa, Asia, Eastern Europe and Latin America found that over one-third of women discontinued their contraceptive method by 12 months of use, with 17% - 62% stopping due to method-related reasons including side effects [11]. Discontinuation is generally higher for hormonal contraceptives, for short-acting methods, and among younger women [11–13]. Evidence suggests that the availability of a broader range of contraceptive methods is associated with lower discontinuation by making it easier for women to switch to a different method if they are dissatisfied with their original choice [14, 15]. Although research on the specific association between contraceptive service provision and discontinuation is mixed [16], an analysis of DHS data from 15 countries found that 7%-27% of women discontinued their contraceptive method in the first year for reasons related to the quality of the service delivery, most of which could be addressed through quality improvement activities [17]. Overall, the evidence suggests that service quality and contraceptive continuation are associated [15, 17, 18], but no research has addressed the specific needs of women in unstable or conflict settings and how best to support them to continue contraceptive use and avoid unintended pregnancies.

### Context and program description

Two decades of war and instability in the eastern region of the Democratic Republic of the Congo (DRC) have resulted in a compromised health system. The population of the province of North Kivu continues to be exposed to high levels of violence and displacement [19]. DRC has the sixth highest maternal mortality ratio in the world at 730 maternal deaths per 100,000 live births [20], and is characterized by low contraceptive use. The 2014 DHS found that modern contraceptive prevalence was 7.8% nationally, 11.6% in North Kivu and 4.6% in rural areas, with male condoms the most commonly used modern method [21]. Use of long-acting and permanent contraceptive methods was very low at 1.7% nationally, 5.5% in North Kivu and 1.1% in rural areas. Unmet need for contraception in North Kivu is 37.5%, and 28.8% of women nationally reported an unintended pregnancy in the previous five years. Since 2012, the DRC government has made a greater commitment to improve contraceptive services; this has yet to diffuse to the provincial level [22].

Save the Children, in collaboration with Columbia University’s Reproductive Health Access, Information and Services in Emergencies (RAISE) Initiative [23], began supporting the Ministry of Health (MOH) to provide contraceptive services and post-abortion care in 34 public health facilities in three rural health zones of North Kivu, DRC in July 2011, and in five facilities in Goma, the provincial capital, in November 2012. Short- and long-acting reversible contraceptive methods (pills, injectables, condoms, intra-uterine devices (IUDs) and implants) were introduced at health centers and hospitals. Training on permanent methods in 2015 made male and female sterilization more available at the hospitals. All contraceptive methods were provided free of charge in the supported facilities.

Program support to the health facilities addressed Bruce’s fundamental elements of quality for contraceptive services: availability of a range of short- and long-acting methods, competency-based clinical training for providers, training on contraceptive counselling, provision of essential equipment and supplies and improved monitoring and evaluation [24]. Mechanisms to improve continuation of contraceptive method use and follow-up of short-acting method users were put in place. Once the services were established, the program expanded its focus on quality improvement. Two in-depth program reviews were conducted by RAISE staff to identify areas needing improvement and to help program and MOH staff develop ways of addressing them.

The program provided essential equipment, supplies and contraceptives to the facilities, primarily through the health zone MOH office. Nurses were trained to provide short- and long-acting contraceptives to ensure their availability at health center level. All clinical training organized by the program was competency-based, meaning each provider practiced on anatomic models and live clients under observation by skilled trainers until competence was attained.

The program focused on introducing high quality contraceptive services and helping clients to start a method. However, they recognized that it is also important to support women to continue use of the contraceptive method that best suits them. Understanding the reasons clients discontinue or continue contraceptive methods can help improve programs and ultimately contribute to helping them to effectively plan their families. Therefore, the program conducted an evaluation to measure 12-month contraceptive continuation.

## Methodology

### Study design and sample

**Fig 1:**
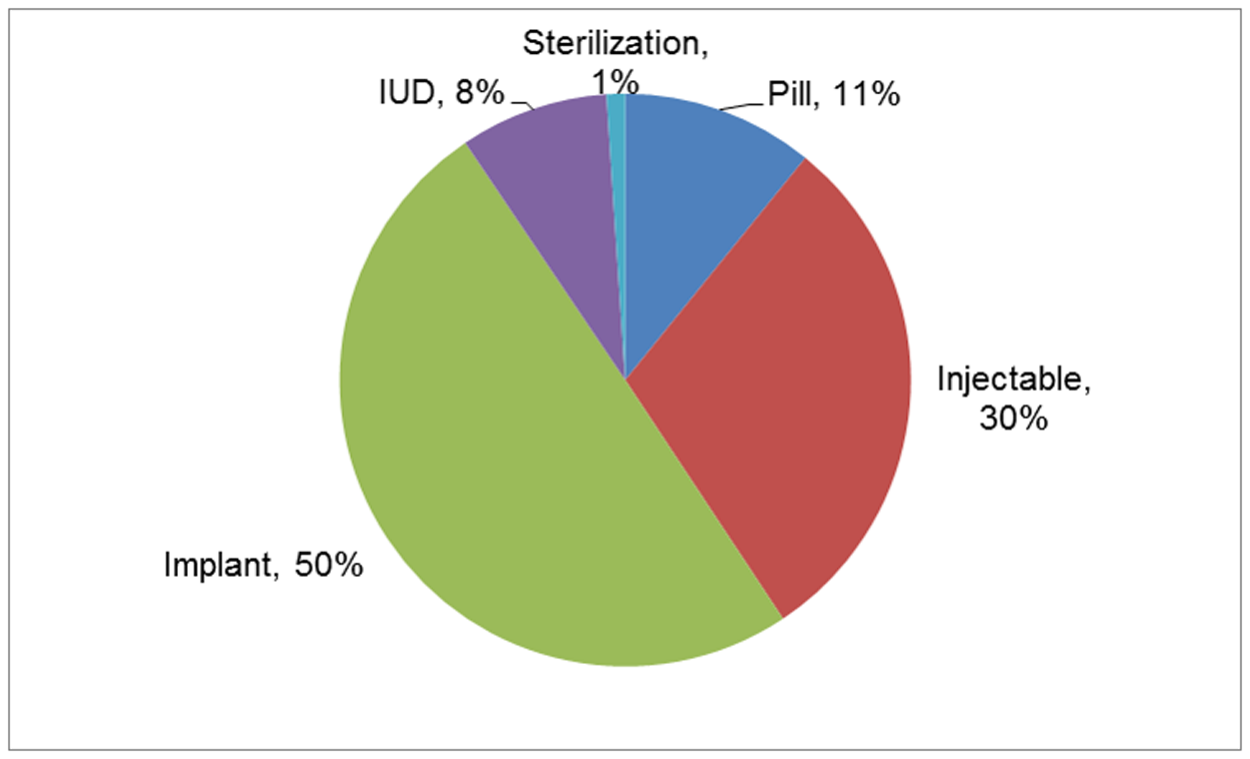
Distribution of contraceptive method initiations, Save the Children program, DRC, Jun 2011-Dec 2015. This represents contraceptive method initiations rather than individual women.

This evaluation used retrospective data from Save the Children-supported MOH health facilities in Mweso and Masisi health zones, DRC. More than 50,000 modern contraceptive initiations occurred between June 2011 and December 2015 at the 34 supported MOH facilities; 58% initiated a long-acting reversible contraceptive (LARC): IUD or implant (Fig 1). Nine Save the Children-supported MOH facilities were selected for this evaluation because most of the villages in their catchment areas were accessible during rainy season when the survey was conducted and security was sufficient for the interviewers to safely travel and locate the clients. A sample of women, aged 18 or older, who initiated a short-acting (pills, injectables) or long-acting (IUDs, implants) contraceptive method 12–18 months prior to data collection was drawn from the registers of these facilities. A sample size of 211 short-acting method acceptors and 306 LARC acceptors was selected to detect (with 95% confidence and 80% power) a 13% difference between short-acting and LARC acceptors based on documented contraceptive discontinuation at 12 months [25–27]. We used stratified systematic sampling with different selection fractions for each method to ensure a minimum of 100 clients for each contraceptive method. Contraceptive acceptors from June through August 2014 (May through August for IUD clients) were identified. Data on the contraceptive method the woman chose, the date she started the method, her name, age and village name were extracted. We extracted a sample from the registers larger than needed to account for anticipated difficulties locating women for interviews given the unstable population and far distances. Women whose village was outside the catchment area of the facility, whose village was largely inaccessible or for whom the information in the register was insufficient to locate them were then deleted from the sampling frame.

Once data collection began, new militia movements prevented access to one facility and its catchment area. In addition, the use of community health workers (CHWs) to locate clients was found to be infeasible in the urban location of three selected facilities. Four additional rural facilities were selected to replace these four facilities. Because these facilities had lower client loads than the original four, the team extracted all clients who started one of the four contraceptive methods from April through September 2014 (implants for May through August only). Due to the lower client numbers in the replacement facilities, the team returned to the other facilities already included and extracted client data from additional months: all IUD, pill and injectable clients from April and May, and all contraceptive starters for September 2014.

### Study procedures

The 87-question quantitative survey questionnaire included topics such as demographic characteristics; birth history; current reproductive intentions; partner involvement in contraceptive decision-making; date of, experience with and reasons for discontinuation and/or switching of methods; future intention to use for discontinuers; and continuers’ experience of problems with their method. Women were asked about the quality of the information received at contraceptive method initiation including if the provider explained how to use the method, possible side effects, what to do in case of side effects and responded to all of her questions. Questions were also asked about satisfaction with the services received at time of contraceptive initiation and at any later point (e.g., if she returned because of problems with the method). Questions were taken from pre-existing French sources for this study [28, 29] whenever possible with adaptations of the response options for the local context. The questionnaire was developed in French, and then translated into two languages: Congolese Swahili and Kinyarwanda. These translations were then reviewed by the study team for accuracy. The questionnaire was piloted among clients not included in the study sample.

The study team worked with the providers at the facilities and affiliated CHWs to locate the selected women. CHWs or nurses visited the selected women in their homes to ask if they were willing to discuss their use of health services at the facility with an interviewer. According to MOH policy and current practice, these CHWs already visit women in their homes to discuss their health care and remind them of follow-up appointments for contraception so it was not unusual for the CHWs to visit women at home. The CHWs were told that the women were selected as clients of the health facility, not as contraceptive acceptors, to maintain their confidentiality. Once a woman agreed to participate, a trained female interviewer interviewed her in private. Women who lived more than a one hour walk from the facility were invited to an interview at the facility or at a location mid-way between. These women who were interviewed at a location outside their villages were given a sachet of salt and a bar of soap to reimburse their travel time. Data collection took place in October and November 2015.

### Ethical considerations

Ethical approvals for the study were obtained from the Institutional Review Board (IRB) of the Mailman School of Public Health, Columbia University, the Ethical Review Committee of Save the Children and the North Kivu Provincial MOH. Interviewers read a consent script to the client and obtained her verbal consent before commencing the interview. If the client indicated that she did not wish to participate, she was thanked for considering involvement in the study and no further questions were asked of her. Participant names were not entered on survey questionnaires to preserve anonymity, and records used to locate the client were destroyed once she was located. A waiver of written consent was obtained from the IRB to preserve participant anonymity. The program intervention is funded by an anonymous private foundation. The donor had no role in study design, data collection, analysis or interpretation of the findings.

### Statistical analysis

Data were entered into CSPro 6.0 and subsequently exported to PASW (SPSS) Version 23 for cleaning and analysis. Discontinuation is defined as women reporting that they stopped continuous use of their baseline contraceptive method within 12 months of accepting the method. Two women who were missing dates for discontinuation were excluded from the analysis. Key socio-demographic characteristics of short-acting method versus LARC acceptors were compared using chi-square statistics for categorical data and t-tests for continuous data. Where significant differences were found, the characteristics were compared by contraceptive method using Pearson chi-squares or one-way analysis of variance. Kaplan-Meier estimates of the probability of discontinuation stratified by type of baseline contraceptive method (short-acting or LARC) were plotted; the Wilcoxon (Breslow) test was used to assess differences in the survival curves. Censoring occurred when the woman reported that she stopped continuous use of her baseline contraceptive method.

Unadjusted and adjusted Cox proportional hazard ratios were estimated to assess factors associated with time to discontinuation. Factors commonly associated with discontinuation were added to the adjusted hazards model. These factors included type of baseline contraceptive method (short-acting/LARC), age and parity (continuous variables), displacement in the past year (yes/no), education (none or some primary/completed primary or higher), marital status (not married/married or living with partner) and desire for more children (wanted children within two years, wanted children after two years or wanted no more children). The proportional hazards assumption was investigated and no time variation was detected. To assess possible problems of collinearity, separate analyses were run with parity and desire for more children; because the results showed no significant differences, the results here present both variables.

## Results

A total of 548 contraceptive acceptors was interviewed (representing 70% of the women sought or invited to come for an interview): 304 who started a short-acting method and 244 who started a LARC in the 12–18 months prior to the interview. Table 1 shows that LARC acceptors were slightly older than short-acting method acceptors (mean age 28.9 versus 27.5, p = .009). Half of LARC acceptors (50.4%) reported having been displaced at least once in the previous year compared to 36.5% of short-acting method acceptors (p = .001). No other socio-demographic differences were found among short- and long-acting contraceptive acceptors. In the overall sample, most women were married or in union (82.7%) and half had no formal education (51.1%). Mean gravidity for the women was 5.3 with a mean of 4.2 living children. The vast majority of respondents wanted no more children (44.3%) or to wait more than two years for their next pregnancy (36.3%). Women who accepted IUDs at baseline were older (mean age 30.6 years compared to 27.4–27.7 years for the other method acceptors, p < .001), more likely to have been displaced in the past year (60.4% compared to 27.6% - 43.4%, p < .001) and had higher mean gravidity (6.0 compared to 4.9–5.3, p = .05) than other contraceptive method acceptors.

**Table 1:** Characteristics of respondents, by baseline contraceptive method type.

	Total acceptors (N = 548) %(n)	Short-acting acceptors (n = 304) %(n)	LARC acceptors (n = 244) %(n)	p-value
**Age (years)**				
18–24	32.5% (178)	36.2% (110)	27.9% (68)	p = .017*
25–34	48.5% (266)	48.7% (148)	48.4% (118)	
35–49	19.0% (104)	15.1% (46)	23.8% (58)	
**Mean age (SD), years**	28.1 (6.3)	27.5 (6.1)	28.9 (6.5)	p = .009*
**Displaced in last year (yes)**	42.7% (234)	36.5% (111)	50.4% (123)	p = .001*
**Marital status**				p = .938
Married, living with husband	82.7% (453)	82.2% (250)	83.2% (203)	
Married, not living with husband	3.3% (18)	3.6% (11)	2.9% (7)	
Not married, living with partner	1.8% (10)	1.6% (5)	2.0% (5)	
Not married, not living with partner	12.2% (67)	12.5% (38)	11.9% (29)	
**Religion**				p = .598
Protestant	43.8% (240)	45.1% (137)	42.4% (103)	
Catholic	16.1% (88)	16.1% (49)	16.0% (39)	
Adventist	20.8% (114)	21.1% (64)	20.6% (50)	
Pentecostal/Evangelical	17.0% (93)	16.4% (50)	17.7% (43)	
Other or no religion	2.2% (12)	1.3% (4)	3.3% (8)	
**Formal education**				p = .607
None	51.1% (280)	50.5% (153)	52.0% (127)	
Some primary school	18.4% (101)	17.8% (54)	19.3% (47)	
Completed primary school	11.5% (63)	10.9% (33)	12.3% (30)	
At least some secondary education	18.8% (103)	20.8% (63)	16.4% (40)	
**Gravidity (lifetime pregnancies)**				
0–1	5.5% (30)	6.6% (20)	4.1% (10)	p = .360
2–4	38.9% (213)	40.5% (123)	36.9% (90)	
5–9	46.4% (254)	44.7% (136)	48.4% (118)	
10+	9.3% (51)	8.2% (25)	10.7% (26)	
**Mean gravidity (SD)**	5.3 (3.0)	5.13 (2.934)	5.61 (3.013)	p = .063
**Mean number of living children (SD)**	4.2 (2.1)	4.1 (2.1)	4.3 (2.1)	p = .301
**Desire for more children**				p = .307
Within 2 years	18.1% (99)	20.3% (61)	15.8% (38)	
After 2 years	36.3% (199)	34.6% (104)	39.6% (95)	
Wants no more children	44.3% (243)	45.2% (136)	44.6% (107)	
**Partner is aware of contraceptive use**	79.3% (405)	81.0% (230)	77.1% (175)	p = .333
**Partner approves of contraceptive use**	79.1% (404)	81.0% (230)	76.7% (174)	p = .277
**Decision to start contraception**				p = .624
Joint decision	69.9% (380)	70.4% (212)	69.1% (168)	
Primarily my decision	27.0% (147)	25.9% (78)	28.4% (69)	
Primarily husband/partner	3.1% (17)	3.7% (11)	2.5% (6)	
**Source of information about contraception**				
Health center	78.6% (431)	80.9% (246)	75.8% (185)	p = .179
Community health worker (CHW)	63.1% (346)	59.9% (182)	66.2% (164)	p = .093
Radio	18.1% (99)	18.1% (55)	18.0% (44)	p = 1.00
Husband/partner	7.8% (43)	9.5% (29)	5.7% (14)	p = .138
Friend/family member	13.9% (76)	14.1% (43)	13.5% (33)	p = .933
Community or religious leader	3.5% (19)	3.9% (12)	2.9% (7)	P = .493
**Ever used contraception before**	11.7% (64)	12.5% (38)	10.7% (26)	p = .593
**Quality of information/counselling received from provider**^a^	76.1% (415)	75.2% (227)	77.4% (188)	p = .618
**Complete satisfaction with services received**^b^	93.6% (513)	93.4% (284)	93.9% (229)	p = .976

*Test of significance p < .05

^a^Includes responding yes that the provider explained how to use the contraceptive method, told her about possible side effects, explained what to do in case of side effects and responded to all of her questions.

^b^Includes being satisfied or very satisfied with the facility’s cleanliness, the providers’ friendliness, the amount of time spent at the facility, the privacy during her time with the provider, the care she received, the respect shown to her by the provider and the expectation that the provider will keep her information secret.

Most women reported that their partner was aware of their contraceptive use (79.3%) and approved (79.1%). Consistent with these results, 69.9% reported the decision to use contraception was made jointly with her partner while 27.0% reported it was primarily her decision. Women reported the health center (78.6%) and community health workers (63.1%) as sources of information about contraception. Before starting their baseline contraceptive, the vast majority of the women (88.3%) had never used contraception. The main reasons given for deciding to begin contraceptive use included wanting to delay pregnancy (85.4%), feeling tired or having health problems (44.9%) or wanting no more children (19.3%). Women reported that the provider discussed other contraceptive methods with them (93.4%), but that they themselves decided which method to choose (89.4%). Overall, 76.1% of women reported that the provider gave sufficient information at time of initiation. Satisfaction with the services received when they started their contraceptive method was very high, with 93.6% reporting complete satisfaction.

**Fig 2:**
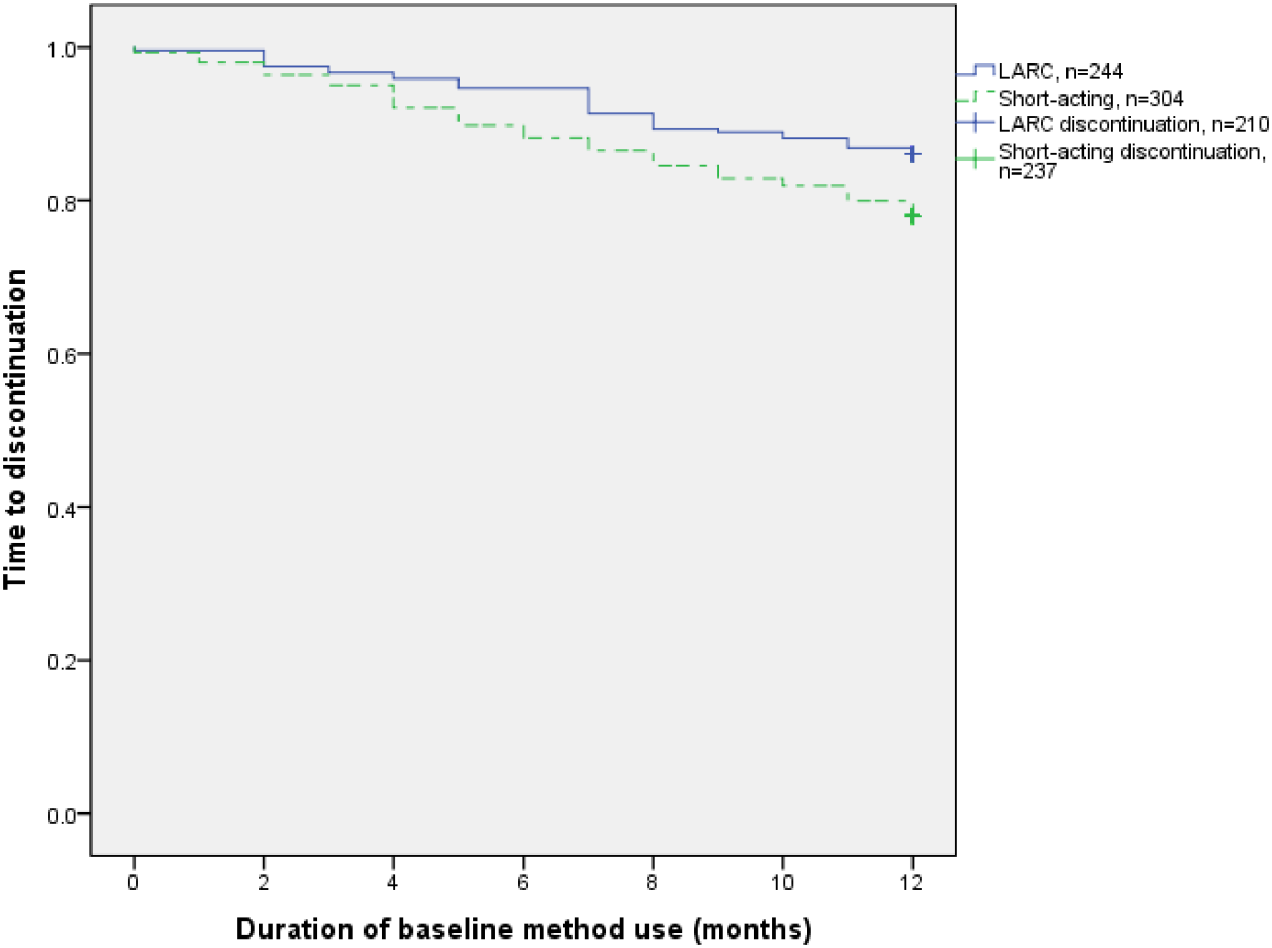
Kaplan-Meier survival curve for 12-month continuation of reversible contraceptives: Short-acting and LARC acceptors, Breslow chi-square p = .015.

At 12 months, 81.6% of women reported using their baseline contraceptive method without interruption, with more LARC acceptors than short-acting method acceptors (86.1% versus 78.0%, p = .02) continuing method use (Table 2). The most common reasons given for discontinuation were that the women desired pregnancy (42.6%) or experienced side effects (32.7%). Only one discontinuer reported stock-outs as the reason for discontinuing contraceptive method use. Fig 2 presents the Kaplan-Meier survival curves, with separate curves for women who started short-acting methods and LARCs (Breslow Wilcoxon test of equality of survivor functions, p = .015); due to the low numbers of discontinuers, median time to discontinuation could not be calculated. Use of a short-acting method was associated with discontinuation within the first 12 months of use (HR 1.74 [95%CI 1.13–2.67]) when adjusted for age, parity, displacement in the last year, education, marital status and desire for more children (Table 3). Of these covariates, only desiring a child within two years (HR 2.58 [95%CI 1.45–4.54]) or after two years (HR 1.9 [95%CI 1.12–3.22]) as compared to wanting no more children were associated with discontinuation.

**Table 2:** Respondents’ baseline contraceptive method discontinuation and switching.

	Total acceptors (N = 548) %(n)	Short-acting acceptors (n = 304) %(n)	LARC acceptors (n = 244) %(n)	p-value
**Baseline method used continuously for 12 months**				p = .02*
No	18.4% (101)	22.0% (67)	13.9% (34)	
Yes	81.6% (447)	78.0% (237)	86.1% (210)	
	**Total acceptors (n = 101)**	**Short-acting acceptors (n = 67)**	**LARC acceptors (n = 34)**	
**Reason for discontinuing baseline method**				
Desired pregnancy	42.6% (43)	44.8% (30)	38.2% (13)	p = .678
Wanted to switch method	28.7% (29)	32.8% (22)	20.6% (7)	p = .292
Side effects	32.7% (33)	29.9% (20)	38.2% (13)	p = .532
Partner/family disliked	5.9% (6)	4.5% (3)	8.8% (3)	p = .669
Became pregnant while using	8.9% (9)	7.5% (5)	11.8% (4)	p = .480
Rumours/method bad for her	5.0% (5)	3.0% (2)	8.8% (3)	p = .332
Other reasons	5.9% (6)	7.5% (5)	2.9% (1)	p = .661
**Switched to another modern method**				p = .998
No	55.4% (56)	55.2% (37)	55.9% (19)	
Yes, in the same month	8.9% (9)	9.0% (6)	8.8% (3)	
Yes, after 1 month	35.6% (36)	35.8% (24)	35.3% (12)	

*Test of significance p < .05

**Table 3:** Unadjusted and adjusted hazard models predicting risk of discontinuation of baseline contraceptive method within first 12 months of use.

	Unadjusted HR (95%CI)	p-value	Adjusted HR (95%CI)	p-value
*Method type*				
Short-acting	1.65 (1.10–2.50)	p = .017	1.74 (1.13–2.67)	p = .012*
LARC	1 (reference)		1 (reference)	
Age (years)	.983 (.953–1.015)	p = .305	.991 (.950–1.04)	p = .687
Displaced in last year (yes)	1.25 (.846–1.85)	p = .263	1.05 (.686–1.60)	p = .832
Parity (no. of lifetime pregnancies)	.995 (.932–1.06)	p = .874	1.07 (.973–1.17)	p = .172
Education (none or some primary)	1.36 (.856–2.17)	p = .191	1.44 (.896–2.33)	p = .132
Marital status (not married)	1.29 (.744–2.23)	p = .367	1.61 (.899–2.9)	p = .109
*Desire for more children*				
Within 2 years	2.33 (1.40–3.89)	p = .001	2.58 (1.45–4.54)	p = .001*
After 2 years	1.62 (1.01–2.59)	p = .045	1.90 (1.12–3.22)	p = .017*
Wants no more children	1 (reference)		1 (reference)	

*Test of significance p < .05

Of the 101 women who discontinued their baseline contraceptive method within 12 months, 55.4% stopped using any modern method for the remainder of the observation period, 8.9% switched during the same month as stopping the previous method and 35.6% switched sometime later during the observation period to another modern method. The majority of the switchers (60.0%) switched to a contraceptive method of the same or similar effectiveness; 28.9% switched to a more effective method and only 11.1% switched to a less effective modern method (Table 4).

**Table 4:** Status of discontinuers (n = 101).

Direction of change in efficacy	Baseline method	Switched to:	n	%
**Discontinued contraceptive use (n = 56)**				
**↓**	Short-acting	No method or traditional method	37	55.4%
	LARC	No method	19	
**Switched to another contraceptive method (n = 45)**				
**↓**	LARC	Short-acting method	5	11.1%
**←→**	Short-acting	Short-acting	17	60.0%
	LARC	LARC	10	
**↑**	Short-acting	LARC	17	28.9%

### Continuing contraceptive users’ satisfaction

One-third (36.8%) of women using their contraceptive method at the time of the interview reported having experienced problems with their method, primarily body or headaches (69.4%) and menstrual changes (72.0%), and most of these women (89.8%) sought care at a facility (Table 5). IUD users were less likely to report any problem (25.0% compared to 28.2%-45.5% among other method users, p = .006), and least likely to report menstrual changes as a problem (45.0% compared to 63.0%-83.1%, p < .003). Most women who sought care (89.8%) reported that the problem was resolved to their satisfaction. Women reported high overall satisfaction with their method (98.6%), and planned to continue their contraceptive use (98.8%). Half of LARC users (52.0%) said they wanted to continue their method for five years or longer, while half (52.5%) of short-acting method users projected using their method for two to four years (p < .001). The majority of IUD users (61.0% compared to 29.4%-46.2%, p < .001) reported wanting to continue method use for five years or more.

**Table 5:** Continuing contraceptive users’ satisfaction^a^.

	All users (N = 429) %(n)	Short-acting users (n = 250) %(n)	LARC users (n = 224) %(n)	p-value
**Experienced problem with current contraceptive method**	36.8% (157)	40.1% (91)	33.0% (66)	p = .130
** Type of problem**				
Head or body aches	69.4% (109)	73.6% (67)	63.6% (42)	p = .180
Nausea or vomiting	18.5% (29)	22.0% (20)	13.6% (9)	p = .184
Menstrual changes^b^	72.0% (113)	82.4% (75)	57.6% (38)	p = .001*
Other problems	5.1% (8)	3.3% (3)	7.6% (5)	p = .229
** Sought care for problem at a health facility**	89.8% (141)	91.2% (83)	87.9% (58)	p = .496
** Problem resolved to her satisfaction**	92.1% (129)	93.9% (77)	89.7% (52)	p = .358
**Satisfied overall with method**	98.6% (421)	99.6% (226)	97.5% (195)	p = .071
**Plans to continue method use**	98.8% (422)	99.1% (225)	98.5% (197)	p = .553
**Projected time to continue current method use**				p < .001*
Less than 2 years	9.2% (38)	12.8% (28)	5.1% (10)	
2–4 years	48.0% (199)	52.5% (115)	42.9% (84)	
5 or more years	42.9% (178)	34.7% (76)	52.0% (102)	
**Projected mean number of years to continue current method use (SD)**	3.7 (2.20)	3.3 (2.0)	4.1 (2.3)	p < .001*

^a^Excludes women who discontinued or switched methods after 12 months and before the interview date.

*Test of significance p < .05

^b^Includes reports that periods stopped, increased or became irregular.

## Discussion

More than 80% of women in this study were still using their baseline contraceptive method at 12 months. While continuation was higher among LARC acceptors than among short-acting method acceptors, as expected, both percentages were higher than commonly found in other studies [27, 30, 31]. Moreover, continuation was similarly high across age, education level and displacement status. This program attracted new users of contraception (88.3% reported no prior contraceptive use) and demonstrated that these new acceptors could also be supported, as needed, to continue contraceptive use as long as they wished. This is the first study of contraceptive continuation in a humanitarian setting; these results provide evidence that effective contraceptive programs that result in initiation and continuation can be successfully implemented in such settings. Further, this evaluation lends support to the association between service quality and contraceptive continuation [15]; the high percentages of women continuing contraceptive use found here indicate that delivering high quality contraceptives services in humanitarian settings is possible. As previously described, this program addressed Bruce’s six elements of quality of care which appear to enhance contraceptive continuation.

Nearly half of the discontinuers switched to another modern contraceptive method during the study period, although fewer than 10% did so within one month of stopping the baseline method. The program could better assist women who wish to switch contraceptive methods to do so immediately so they are not placed at risk of an unintended pregnancy. Of the discontinuers who switched to a different contraceptive method during the study period, 60.0% changed to a method of similar effectiveness and 28.9% switched to a more effective LARC. This reflects the success the program is having with counseling and making a range of effective short- and long-acting contraceptive methods available to this population.

While LARC acceptors were slightly older and more likely to have been displaced in the last year, few differences in other socio-demographic characteristics between baseline contraceptive method types were found. These data suggest that access to contraceptive methods is equitable and women choose the method they want. Displaced women may be more likely to choose a LARC to avoid the need for resupply visits given the instability of their current living situation. Few women reported previous contraceptive use, reinforcing the notion that when good quality contraceptive services are put in place, women will use them, and may in fact be highly motivated to continue use.

A recent analysis of evidence related to contraceptive discontinuation identified several key programmatic strategies to reduce discontinuation: improving service quality, reducing provider bias and improving technical competence, eliminating stock-outs, increasing access through multiple service delivery options and facilitating task sharing to lower level cadres [15]. As described earlier, the program’s strong focus on quality improvement addressed many of these issues which may therefore have contributed to the high continuation rates. For example, only one woman reported discontinuation due to stock-outs, suggesting the program successfully made the contraceptive methods consistently available. After some initial problems with stock-outs, facility staff received additional training in stock management and forecasting. Although insecurity and poor roads presented challenges to maintaining correct stock levels, stock-outs at the health zone level were minimized.

Mid-level providers (nurses) were trained to provide all short- and long-acting contraceptive methods at health center level, thereby increasing access to them in this rural area. Clinical supervisors provided regular supportive supervision and had good supportive relationships with facility staff with whom they identified problems and solutions together. To ensure technical competence, supervisors observed provider skills twice a year using a checklist and used a database to track provider performance. Clinical supervisors used the data to develop individual support based on each provider’s need. For example, few clients initially chose IUDs. During supportive supervision visits, it was discovered that providers had had little opportunity to practice after their initial training, and therefore lacked confidence in their skills and thus underemphasized IUDs during counselling. Supervisors subsequently carried anatomic models to their supervision visits to observe the providers’ skills and provide coaching. The introduction of post-placental IUDs may have led to increases in IUD acceptance, by adding a new service delivery option, as this method became more familiar to both providers and clients. Making all contraceptive methods available was important as a greater range of available methods increases method choice, and is also associated with lower discontinuation [14].

In addition to improving provider competence, reducing provider bias is also associated with higher continuation [15]. Few women in our sample were under 21 years old or unmarried reflecting program review findings that some providers were uncomfortable providing contraception or specific methods to adolescent and unmarried women. Values clarification activities to discuss attitudes that were unfavourable to good quality care were conducted regularly. Supervisors reinforced the need to treat all clients with respect and offer all women informed choice. The program organized refresher trainings that focused specifically on counselling skills and attitudes to ensure that the providers discussed the range of contraceptive methods from which the woman could choose. The high satisfaction with provider explanations during counselling may reflect the success of these efforts. These results also highlight the need for multi-year donor funding which permitted the program to focus on these quality improvement activities once the basic services were in place.

It is notable that 71.3% of women reported reasons for discontinuing related to a change in their own reproductive intention and not to program weaknesses, i.e., they desired pregnancy or wanted to switch contraceptive methods. Consistent with other studies, side effects were reported by one-third of both short-acting and LARC acceptors as a reason for discontinuation, suggesting a need for additional support to providers to respond to side effects and to improve initial counseling about them [11, 32]. Despite anecdotal evidence from program staff and providers that rumors and misinformation in the community about contraception were problems, only a few women mentioned these as reasons for discontinuation suggesting a successful community education program.

The vast majority of participants reported learning about contraception from program activities. The program trained CHWs as well as satisfied clients, peer educators, community leaders and local community associations to conduct education about contraception in the supported health zones. The broad range of community actors helped to reach men, which may have contributed to the high rates of joint decision-making and partner approval in this population. Community mobilization supervisors visited with the CHWs and provided coaching on communications skills and different strategies to present and discuss key messages. In addition, they helped the program to identify rumors about contraception in the community and respond to them quickly. For those women who did not return for their next set of pills or next injection, the facilities had a system that used CHWs to remind them of their missed appointments. However, during data collection for this study, we found that male CHWs were less helpful finding women using the available information. Female CHWs and providers were more likely to know who their clients were. In DRC, most MOH-affiliated CHWs are male; therefore, the system to promote continuation could be improved, particularly by involving more women as CHWs. To address this gap, Save the Children trained and supported a cadre of female peer educators and satisfied users.

Just over one in three contraceptive users who were using their method at the time of the interview reported experiencing a problem with their method; short-acting method users were more likely to report menstrual changes, especially increased menses, than LARC users. This is consistent with other studies finding high levels of side effects reported by pill and injectable users [11, 15, 33]. Since hormonal contraceptives represent nearly 90% of this program’s method mix, supporting providers to respond to side effects is crucial. The program provided extra coaching to providers on responding to side effects which may have contributed to the low discontinuation as nine in ten women who sought care for their problem reported the problem resolved. Although IUD discontinuers were as likely as other discontinuers to report side effects as a reason for discontinuation of the method, continuing IUD users were least likely to report any problem, and particularly a problem with menstrual changes, compared to the other method users. This suggests that if women are supported through the early side effects after IUD insertion, they end up reporting fewer problems with their method later on.

The high percentage (over 80%) of women who did not want to become pregnant within two years likely also contributed to the high continuation. This program has successfully integrated LARCs into their method mix, and clients are highly satisfied with the methods. It is, however, notable that more than one in three short-acting method users said they wished to continue contraceptive use for five or more years. In this rural area with far distances, poor or non-existent roads, high insecurity and frequent displacement, these women may be interested in switching to a LARC that does not require return visits to the facility every three months. Further, nearly half of respondents reported wanting no more children, making them potential candidates for sterilization. Now that sterilization is more available, the program should improve education about permanent methods for those who are finished with child bearing. Good quality services that ensure the availability of a range of short-acting, long-acting and permanent contraceptive methods and encourage immediate switching among those who are dissatisfied with their method allow women to exercise their reproductive choice.

### Limitations

Due to insecurity and poor road access, a subset of supported facilities was identified from which clients were selected rather than selecting the sample from all supported facilities. Clients from outside the catchment areas of the facilities were excluded; clients who lived far from the facilities are underrepresented as they were more difficult to locate for interviews and less likely to come when invited for an interview. Additional clients could not be found because they had moved or traveled, or the information in the register was insufficient for the CHWs to identify them. The sample is therefore not representative of all clients starting a contraceptive method at Save the Children-supported facilities in North Kivu. The lack of a comparison group makes it difficult to conclude the reasons for the high continuation found here. Continuation may be overestimated in our sample as a woman could have reported continuous use of pills or injectables even if she returned late for a subsequent dose. In addition, those who lived furthest away from the facility, and may therefore have more difficulty returning for the next dose of a short-acting method, are underrepresented. Due to low client numbers, clients from more than one month were selected to ensure sufficient sample size, meaning that women were interviewed 13–19 months after beginning their method to ensure they had the opportunity to use it for at least 12 months at the time of the interview. The data are therefore subject to recall bias: women may have misremembered the timing or the circumstances surrounding their starting, stopping or switching a contraceptive method. A retrospective study, however, was the most feasible study design given the instability and insecurity in this setting as well as the funding and time available. Courtesy bias may have affected the responses to the service quality questions.

## Conclusion

This study, the first to examine contraceptive continuation in a humanitarian setting, demonstrates that women can be supported to continue use of their desired contraceptive method, even in a difficult, unstable and low prevalence setting like North Kivu, where 82% of our sample continued method use at 12 months. A focus on service quality and efforts to address provider skills and ensure consistent availability of commodities likely contributed to the high rates of contraceptive use and continuation. This highlights the need for multi-year funding in humanitarian settings to enable this emphasis on quality improvement. While the program has strengthened the health system in the province, the DRC health system is weak and continued donor support will be required in the future to ensure sustainability. When good quality services, with a choice of short- and long-acting contraceptives, are in place, women will not only choose to start, but also continue, to use contraception to exercise their right to reproductive choice.

## Supporting information

S1 File Survey dataset. (XLSX)
